# PKN2 enhances the immunosuppressive activity of polymorphonuclear myeloid-derived suppressor cells in esophageal carcinoma by mediating fatty acid oxidation

**DOI:** 10.1186/s10020-025-01132-6

**Published:** 2025-03-11

**Authors:** Xiao Fu, GuoQing Zhang, ZhiChao Hou, TingTing Fu, GuangHui Cui

**Affiliations:** 1 Department of Thoracic Surgery, The First Affiliated Hospital of Zhengzhou University, No.1 East Jianshe Road, Zhengzhou, 450052 China; 2 Department of Respiratory Medicine, The First Affiliated Hospital of Zhengzhou University, Zhengzhou, 450052 China

**Keywords:** Myeloid-derived suppressor cells, PKN2, Fatty acid oxidation, Organoid, Immunosuppressive activity

## Abstract

**Background:**

Myeloid-derived suppressor cells (MDSCs) in tumor microenvironment reduce the efficacy of immunotherapy. PKN2 plays a role in colon cancer, but its function in esophageal cancer (EC) remains unclear. This study investigated PKN2 expression in MDSCs derived from EC tissues and determined whether PKN2 regulates immunosuppressive activity of MDSCs by mediating fatty acid oxidation (FAO).

**Materials and methods:**

PKN2 expression was determined in GEO database, EC patients, and 4-NQO-induced EC mice, as well as in different types of immune cells. The effect of PKN2 on the function of polymorphonuclear myeloid-derived suppressor cells (PMN-MDSCs) was investigated by co-culture of PMN-MDSCs and CD4^+^/CD8^+^ T cells. The co-culture of patient-derived organoids and autologous immune cells was performed to observe the effect of PKN2 on the immunosuppressive function of PMN-MDSCs.

**Results:**

PKN2 is highly expressed in EC tumor tissues compared to normal tissues, especially in tumor-infiltrated PMN-MDSCs. Overexpressing PKN2 in PMN-MDSCs contributes to the immunosuppressive activity of PMN-MDSCs in vitro. PKN2-overexpressing PMN-MDSCs inhibited the killing ability of cytotoxic T lymphocytes and promoted EC organoid growth. PKN2 promotes FAO in PMN-MDSCs via CPT1B (a key enzyme of FAO). Mechanistically, PKN2 promotes CPT1B transcription by upregulating STAT3 phosphorylation.

**Conclusions:**

PKN2 expression was increased in PMN-MDSCs derived from human and mouse EC tissues. PKN2 plays a role in enhancing the immunosuppressive activity of PMN-MDSCs by facilitating STAT3 phosphorylation and CPT1B transcription, which in turn leads to increased CPT1B-mediated FAO in PMN-MDSCs. Targeted inhibition of PKN2 is expected to improve immunotherapeutic efficacy in EC patients.

**Supplementary Information:**

The online version contains supplementary material available at 10.1186/s10020-025-01132-6.

## Introduction

Esophageal cancer (EC) represents a significant malignant tumor with considerable potential to negatively impact human health. The pathological types of EC can be broadly classified into two main categories: esophageal squamous cell carcinoma (ESCC) and adenocarcinoma. ESCC is the most prevalent pathological type, particularly in the upper two-thirds of the esophagus, whereas adenocarcinoma is more common in the lower one-third region of the esophagus (Deboever et al. 2024). As indicated by data published by the National Cancer Center of China, the estimated number of new cases of EC in China in 2022 is approximately 253,000, with an estimated 194,000 deaths (Han et al. 2024). Consequently, EC persists as a significant public health concern in China.

In recent years, the advent of immunotherapy has precipitated a transformation in the approach to treating esophageal cancer (Kadono et al. 2024). Immunotherapy has become a fundamental component of EC treatment regimens, resulting in improved survival outcomes for patients. For example, the ASTRUM-007 study demonstrated that the combination of serplulimab and chemotherapy resulted in a notable prolongation of both progression-free survival and overall survival compared to placebo in the first-line treatment of PD-L1-positive locally advanced and metastatic unresectable ESCC (Li et al. 2024; Gao et al. 2024). It is important to note, however, that not all patients with EC respond to immunotherapy. A more profound comprehension of the mechanisms responsible for the inefficacy of immunotherapy in EC could facilitate the expansion of the patient population benefiting from this approach and enhance the success rate of immunotherapy.

The accumulation of a substantial number of immunosuppressive cells, particularly myeloid-derived suppressor cells (MDSCs), within the tumor microenvironment represents a significant impediment to the efficacy of tumor immunotherapy (Xu et al. 2024). MDSCs are characterized by their immunosuppressive properties, which primarily affect T cells. MDSCs can impede the induction and activation of T cells through a multitude of mechanisms and pathways, thereby inhibiting their capacity to kill tumor cells (Zeng et al. 2024). A substantial body of evidence indicates that the accumulation of MDSCs is closely associated with clinical outcomes, anticancer therapy, and drug resistance in tumor patients (Kapor et al. 2024). Therefore, the targeting of MDSCs represents a pivotal aspect of tumor therapy. The current methods of targeted intervention against MDSCs are focused on three main components: the promotion of MDSC maturation and differentiation, the reduction of local MDSC numbers, and the inhibition of MDSC function. However, the role of MDSCs in EC remains unclear.

Studies have underscored the pivotal role of energy metabolic pathways in the differentiation and function of MDSCs. Alterations in myelopoiesis, MDSC maturation, and function may be dependent on enhanced central carbon metabolism and an increase in cellular bioenergetic status. For example, 2-deoxyglucose has been demonstrated to prevent monocytic MDSCs (M-MDSCs) from undergoing anterior somatic differentiation by inhibiting glycolysis (Wu et al. 2016). Conversely, it was demonstrated that metformin-promoted glycolysis significantly rescued the rapamycin-induced deficiency of M-MDSCs. Furthermore, a notable increase in fatty acid oxidation (FAO) was observed in tumor-infiltrating MDSCs (Wang et al. 2024a, b; Mohammadpour et al. 2021). An increase in fatty acid uptake and activation of FAO, along with an increase in mitochondrial mass, resulted in the up-regulation of key enzymes of the oxidative phosphorylation pathway and an increase in oxygen consumption, as observed in a mouse tumor tissue model. The combination of low-dose chemotherapy with the inhibition of FAO was observed to significantly suppress the immunosuppressive effects of MDSCs in tumor tissues, thereby inducing a significant anti-tumor effect (Hossain et al. 2015). It would be beneficial to determine whether FAO-mediated MDSC regulation plays a role in EC progression.

Protein kinase N (PKN) is a type of serine/threonine protein kinase that shares structural domains with members of the protein kinase C (PKC) family and is therefore a member of the PKC superfamily (Marrocco et al. 2019). Three principal isoforms of PKNs have been identified, which are PKN1, PKN2, and PKN3. The involvement of PKN2 in the regulation of intracellular glycolipid metabolism, cell cycle and polarity, as well as tumorigenesis and development, has been demonstrated (Murray et al. 2022; Li et al. 2021; Liu et al. 2021). It has been demonstrated that PKN2 can influence the progression of colon cancer by regulating macrophage polarization in tumor immunity (Cheng et al. 2018). The function of PKN2 in EC remains unclear. The objective of this study was to examine the expression level of PKN2 in MDSCs derived from human and mouse EC tissues and to ascertain whether PKN2 regulates the immunosuppressive activity of MDSCs by mediating FAO.

## Materials and methods

### GEO datasets

The validation cohorts, consisting of complete expression profile data (GSE20347), were obtained from the GEO database (https://www.ncbi.nlm.nih.gov/gds). The datasets include 17 normal esophageal tissues and 17 ESCC tissues.

### Clinical samples

Surgical samples, including tumor tissues and paired paracancer tissues, were collected from 40 esophageal cancer patients, who were admitted to The First Affiliated Hospital of Zhengzhou University from January to November of 2023.

### Immunohistochemistry of tissues

Immunohistochemistry of ESCC tissues and adjacent tissues was performed using anti-PKN2 (1/50, ab314021, Abcam, Cambridge, UK) antibody as previously described (Chen et al. 2024).

### Isolation of macrophages, B cells, monocytes, plasma cells, and MDSCs from mice

C57BL/6 male mice aged 6–8 weeks were purchased from Changzhou Cavens Laboratory Animal Co. (Changzhou, Jiangsu, China). The mice were administered 1 mL of 3% mercaptoacetate broth via intraperitoneal injection once daily for a period of three consecutive days. Following a three-day period, the mice were euthanized. The detailed description of macrophage isolation can be seen in supplemental materials.

Mouse blood was collected from healthy mice and lymphocytes were isolated from blood samples using the Mouse Peripheral Blood Lymphocyte Separation Kit (No. C0029S, Beyotime, Beijing, China). Subsequently, mouse B cells were isolated from lymphocytes using magnetic-activated cell separation (MACS). The detailed description of mouse B cell isolation can be seen in supplemental materials.

Mouse monocytes were isolated from blood using the EasySep™ Mouse Monocyte Isolation Kit (No. 19861, STEMCELL Technologies Inc., Vancouver, BC, Canada), following the manufacturer’s instructions.

Mouse plasma cells were isolated from bone marrows using the CD138^+^ Plasma Cell Isolation Kit (No. 130-092-530, Miltenyi Biotechnology, Bergisch Gladbach, Germany), following the manufacturer’s instructions.

MDSCs were isolated from mouse bone marrows. The detailed description of mouse MDSC isolation can be seen in supplemental materials.

### Quantitative real-time PCR (qRT-PCR)

Total RNA was isolated using TRIzol reagent (Invitrogen, Waltham, MA, USA), in accordance with the protocol specified by the manufacturer for tissue samples. Subsequently, cDNA was synthesized from total RNA using the QuantiTect Reverse Transcription kit (Qiagen, Manchester, UK) in accordance with the manufacturer’s instructions. To assess the mRNA expressions, qRT-PCR was performed using the IQ SYBR Green Supermix (Bio-Rad, Hercules, CA, USA) and analyzed on the IQ5 Thermocycler (Bio-Rad, Hercules, CA, USA). The mRNA expressions were normalized to that of the housekeeping gene, GAPDH, with qRT-PCR assays performed in triplicate. The sequences of the primers utilized in qRT-PCR are provided in supplemental materials. Gene expression levels were determined using the 2^−ΔΔCt^ method to calculate fold changes, where ΔΔCt is calculated as ΔCt of the sample minus ΔCt of the control, with ΔCt being the cycle threshold (Ct) value of the target gene minus that of the housekeeping gene.

### Flow cytometry

Flow cytometry analysis was conducted utilizing either a BD FACS Celesta or a FACS Fortessa X20 instrument. The resultant data were processed with the FlowJo software, versions 10.9.1, provided by Tree Star in Ashland, Oregon, USA. For immunostaining, a set of antibodies was employed in accordance with the manufacturer’s guidelines: anti-CD45, anti-CD33, anti-CD14, anti-CD15, anti-CD11b, anti-PKN2 (ab314021), all sourced from (Abcam, Cambridge, UK). Post-staining, the cells were incubated for a duration of 20 min at 37℃ in an atmosphere containing 5% CO_2_. They were then rinsed twice with a flow cytometry buffer composed of PBS supplemented with 2% FCS, prior to being analyzed by FACS. Myeloid cells were identified as CD45^+^CD11b^+^, M-MDSCs as CD45^+^CD11b^+^CD33^+^CD14^+^, and polymorphonuclear myeloid derived suppressor cells (PMN-MDSCs) as CD45^+^CD11b^+^CD33^+^CD15^hi^.

For immunostaining of ESCC mouse model, anti-CD45, anti-CD11b, anti-Ly6G, anti-Ly6C, F4/80, CD19, CD11c (all from Abcam), and anti-PKN2 (MA5-15887, Invitrogen, Waltham, MA, USA) were used. Myeloid cells were identified as CD45^+^CD11b^+^, M-MDSCs as CD11b^+^Ly6G^−^Ly6C^+^, TAMs as CD45^+^CD11b^+^F4/80^hi^, B cells as CD45^+^CD19^+^, DCs as CD45^+^CD11b^low^CD11c^hi^, and PMN-MDSCs as CD11b^+^Ly6C^−^Ly6G^+^.

### Establishment of spontaneous EC mouse model

Following a one-week period of acclimation, the C57BL/6 male mice aged 4–8 weeks were provided with drinking water that included 100 µg/mL of 4-nitroquinoline-1-oxide (4-NQO) for 16 weeks. Mice in the control group were provided with normal water. Throughout the study, the mice’s activity levels, body weights, food and water consumption, and survival status were monitored and documented at two-day intervals. Esophageal tissues and spleen tissues were collected at the 30th week.

### Establishment of xenograft mouse model

Mouse esophageal cancer cells AKR (SNL-647, Procell Technology, Wuhan, Hubei, China) were injected subcutaneously [1 × 10^5^ cells, and matrix gel mix (1:1)] into C57/BL mice (male, 6–8 weeks old, 17–20 g). One week later, PMN-MDSCs (2 × 10^6^ cells, infected with PKN2 overexpressing lentivirus and negative control vector, respectively) were injected intratumorally into the tumors of 5 mice per group. The volume of subcutaneous tumors was measured and recorded every 3 days, and tumor tissue was collected by executing the mice on day 15. Subcutaneous tumor tissues were subjected to immunofluorescence staining using CD8A (CD8^+^ T marker) antibody.

### Western blotting

A total of approximately 30 µg of protein samples were subjected to sodium dodecyl sulfate-polyacrylamide gel electrophoresis (SDS-PAGE) on a 10% gel. Subsequently, the proteins were transferred onto polyvinylidene difluoride (PVDF) membranes (Millipore, Temecula, CA, USA). Subsequently, the membranes were incubated for one hour at room temperature with 10% non-fat dry milk in TRIS-buffered saline with 0.5% Tween 20 (TBST). Subsequently, the samples were incubated with specific primary antibodies at 4 ℃ overnight. The antibodies utilized were listed in supplemental materials. Subsequently, the membranes were incubated with secondary antibodies for an additional hour at room temperature, diluted at a ratio of 1:5000 in TBST. The immunoblots were detected using an enhanced chemiluminescence (ECL) kit sourced from Pierce in Shanghai, China. The bound antibodies were visualized using the FluorChem FC3 system from ProteinSimple in the USA, and the band densities were quantified with the AlphaView software, version 3.4.0.0. The intensities of the proteins were normalized against β-tubulin for ratio calculation.

### Detection of T cell proliferation by the co-culture system

PMN-MDSCs were isolated from mouse EC models using magnetic bead sorting. The cells were transfected with PKN2 overexpression lentivirus as well as negative control (NC) lentivirus (RiboBio Technology, Guangzhou, Guangdong, China). CD4⁺ and CD8⁺ T cells were obtained from mouse spleen by magnetic bead sorting and labeled with CFSE (C34554, Invitrogen). Approximately 24 h prior to the commencement of the co-culture period, which was set at 72 h and involved a 1:1 ratio of PMN-MDSCs to T cells, the former were transfected with lentivirus. The co-culture system was supplemented with anti-CD3 (10 µg/mL, 100253, BioLegend, San Diego, CA, USA) and anti-CD28 (5 µg/mL, 102121, BioLegend) to activate T cells, whereas the NC group was not supplemented with anti-CD3 and anti-CD28. A flow cytometry assay was utilized to assess T cell proliferation.

### Enzyme-linked immunosorbent assay (ELISA) assay

IL-2 (ab242470, Abcam), IFN-γ (EK0375, Boster Biological Technology, Wuhan, Hubei, China), TGF-β (ab119557, Abcam), and IL-10 (EK0417, Boster Biological Technology) were measured in the cell culture supernatants using a mouse ELISA kit in accordance with the manufacturer’s instructions.

### Detection of ROS level

ROS level was detected using the Reactive Oxygen Species Assay Kit (S0033S, Beyotime, Beijing, China). Fluorescence intensity was determined using flow cytometry and FlowJo Software, versions 10.9.1.

### FAO modulation detection

The Fatty Acid Oxidation (FAO) Assay Kit (catalog number BR00001) was utilized to determine the FAO level. The FAO reaction was initiated and terminated in accordance with the instructions provided by the supplier, Assay Genie. Subsequent to the initiation and termination of the FAO reaction, the optical density (OD) at 492 nm was measured using a plate reader.

### Level of acetyl-CoA detection

The level of acetyl-CoA was detected using the Acetyl Coenzyme A Content Assay Kit (catalog number AKFA019U-1; Boxbio, Beijing, China). The assay was performed in strict accordance with the manufacturer’s instructions.

### Establishment of ESCC organoids

Tumor samples from ESCC patients were treated with a digestion buffer consisting of RPMI 1640 medium with 10% fetal bovine serum (FBS), 1% penicillin-streptomycin, and 4 mg/mL of collagenase (C5138, Sigma-Aldrich, St. Louis, MO, USA). Subsequently, the samples were embedded in Matrigel (Corning Incorporated, Corning, NY, USA). Once the Matrigel had solidified, it was covered with Esophageal Squamous Cell Carcinoma Organoid Complete Medium (ESOM-100, SHR Biotechnology, Wuxi, Jiangsu, China). The organoids were cultured at 37 °C in a CO₂ incubator. The organoids employed in the experiments had reached their 30th passage. Sections of the organoids were subjected to histochemical staining (anti-PanCK and anti-CK5) with hematoxylin and eosin (HE) for the purpose of confirming the tumor status in accordance with the standards set forth by the relevant pathologists.

### Co-culture system of organoids with T cells

CD8^+^ T cells were isolated from healthy individuals using the CD8^+^ T-cell isolation kit (Miltenyi Biotechnology, Bergisch Gladbach, Germany). The cells were pre-stimulated with interleukin-2 (200 units/mL, Peprotech, Cranbury, NJ, USA), anti-CD3 (Peprotech), and anti-CD28 (Peprotech) for a period of 48 h. PMN-MDSCs derived from the same organoid were isolated and transfected with PKN2 overexpression lentivirus or NC lentivirus. On the day preceding the co-culture, CD8^+^ T cells were co-cultured with dendritic cells (DCs) isolated from PBMCs derived from healthy individuals. Subsequently, the ESCC organoids were subjected to co-culture with the CD8 ^+^ T cells and PMN-MDSCs (Cattaneo et al. 2020; Sui et al. 2021; Dijkstra et al. 2018). The ESCC organoids were initially digested into individual cells, and the number of cells was subsequently enumerated. Following CellTrace Violet staining and inoculation into 96-well plates (1 × 10⁴ per well, with the cell number extrapolated from the digested counts), CD8^+^ T-cells (5 × 10⁴ per well) and PMN-MDSCs (5 × 10⁴ per well) were added to the wells for co-culture for 72 h, after which photographs were taken in bright field.

### Detection of oxygen consumption rate (OCR) in real-time

Immunomagnetic beads were used to sort mouse splenic PMN-MDSCs, which were transfected with PKN2 overexpression lentivirus as well as NC lentivirus, and the OCR of the cells was detected after 24 h. Oligomycin (4 µM), FCCP (1.6 µM), rotenone (0.5 µM) + antimycin A (0.5 µM), and etomoxir (18 µM, an FAO inhibitor) were used. The OCR was quantified using a Seahorse XFe96 Analyzer (Seahorse Agilent, USA), with the specific wavelengths for the fluorescent oxygen probes set at 532 nm for excitation and 650 nm for emission. The OCR data were standardized based on the protein content per well, as determined through the use of a BCA assay kit (Monroy-Cárdenas et al. 2024). Each experimental run was conducted with a minimum of three replicates.

### Dual luciferase reporter gene assay

To create the reporter constructs for use in dual-luciferase assays, the promoter regions of CPT1B gene were inserted and assembled into the pGL4.20 plasmid (Promega, Madison, WI, USA). Both the reporter constructs and phRL-TK (internal control) were introduced into the HEK293 cells. The reporter and control plasmids were combined in an equal ratio of 1:1. PKN2 overexpression lentivirus or NC lentivirus were also transfected into cells. After 48 h, the samples were collected for the assessment of the LUC/REN ratio. The dual-luciferase reporter assay system from Promega in the USA was utilized, and the experiments were conducted with three biological replicates.

### Chromatin immunoprecipitation (ChIP)-qPCR assay

The ChIP assay was conducted in accordance with the instructions provided in the EZ-ChIP kit (Millipore, Temecula, CA, USA). In conclusion, PMN-MDSCs were plated at a concentration of 1 × 10⁶ cells per dish and cross-linked using a 1% formaldehyde solution. Subsequently, the cells were harvested and resuspended in a lysis buffer. Subsequently, the cross-linked DNA was subjected to sonication, resulting in the generation of DNA fragments with a length range of 200 to 1,000 base pairs. Subsequently, Protein G agarose was introduced into a diluted DNA buffer and subjected to centrifugation. Subsequently, either an anti-PKN2 antibody or an IgG antibody (Proteintech, Rosemont, IL, USA) was introduced. Subsequently, the protein-DNA complexes were eluted and purified. Subsequently, the purified DNA fragments were subjected to qPCR analysis.

### Statistical analysis

The data are presented as the mean ± standard deviation (SD), derived from three separate experimental trials. Statistical evaluations were conducted using one-way or two-way analysis of variance (ANOVA), with the Bonferroni or Tukey post-test employed for pairwise comparisons. All analyses were conducted using GraphPad Prism version 9 (GraphPad Software, San Diego, California, USA). The Pearson correlation coefficient was employed to examine the relationships between variables. A *P*-value of less than 0.05 was considered statistically significant.

## Results

### PKN2 is highly expressed in PMN-MDSCs of ESCC tumor tissues

**Fig. 1:**
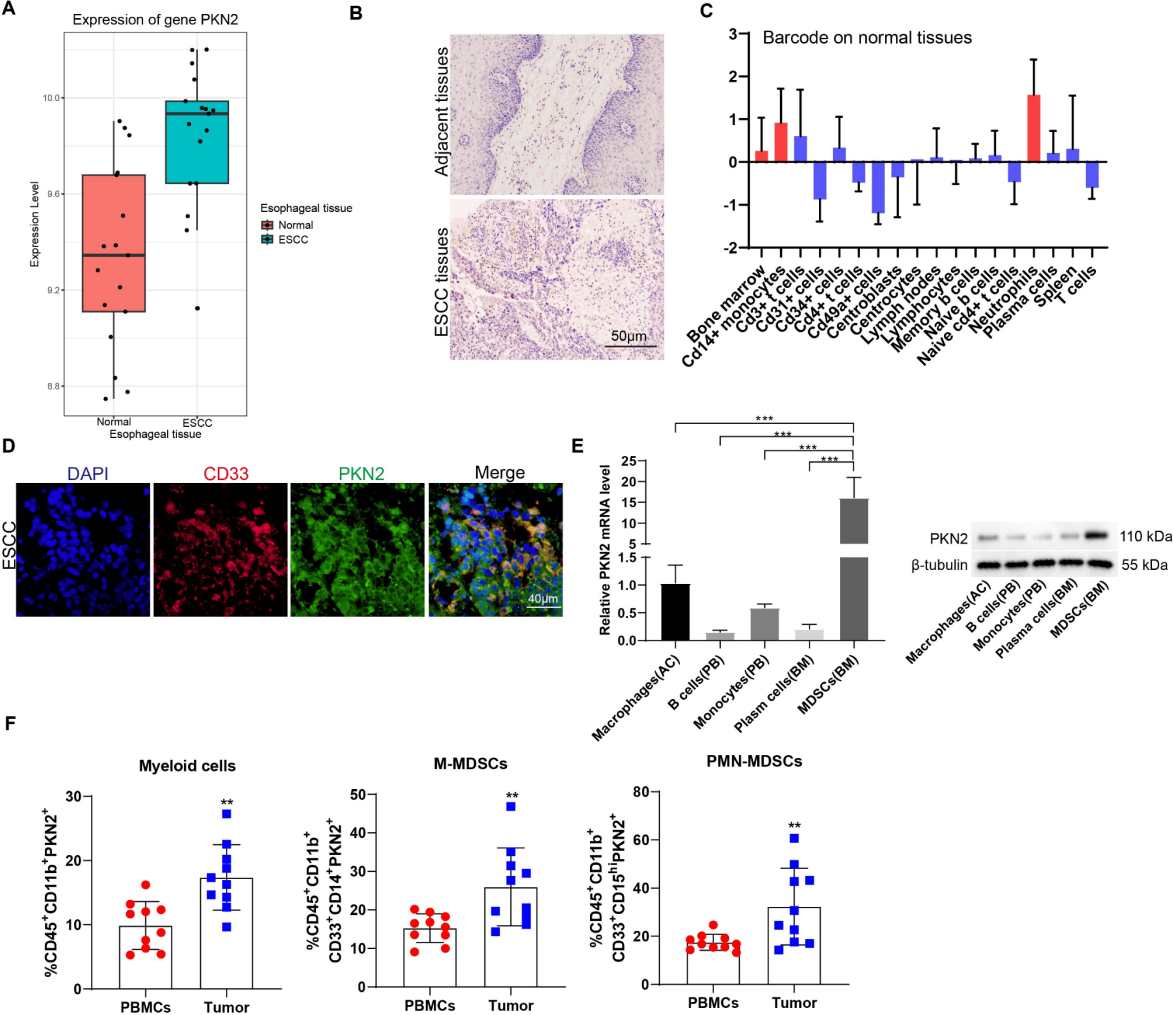
PKN2 is highly expressed in PMN-MDSCs of ESCC tumor tissues. (**A**) The GEO datasets (GSE20347) revealed a significant elevation in PKN2 expression in ESCC tumor tissues (*N* = 17) relative to normal tissues (*N* = 17). (**B**) Immunohistochemistry of PKN2 antibody for EC tissues and adjacent tissues. (**C**) The expression and distribution characteristics of PKN2 in the BioGPS database (http://biogps.org/#goto=genereport_id=5586). (**D**) Immunofluorescence staining results showed that PKN2 was expressed on MDSCs in esophageal cancer tissues. (**E**) The expression of mRNA and protein levels of PKN2 in normal mouse immune cells, including macrophages from abdominal cavity (AC), B cells and monocytes from peripheral blood (PB), and plasma cells and MDSCs from bone marrows (BM), was detected using qRT-PCR and Western blotting (*N* = 3). Statistical analysis: one-way ANOVA with Tukey’s post-hoc test. ***P < 0.001. (**F**) Ten paired PBMCs and tumor tissues were collected from ESCC patients. The percentage of myeloid cells, monocytic MDSCs (M-MDSCs), and polymorphonuclear myeloid derived suppressor cells (PMN-MDSCs) was detected using flow cytometry (*N* = 10). Statistical analysis: two tailed paired student’s t-test. **P < 0.01 vs. PBMCs

The initial step involved a comparative analysis of PKN2 expression between ESCC tumor tissues and normal tissues. The GEO datasets revealed a significant elevation in PKN2 expression in ESCC tumor tissues relative to normal tissues (Fig. 1A). In clinical samples, immunohistochemistry demonstrated that PKN2 expression in tumor tissues was higher than in normal tissues, particularly in stromal cells of tumors (Fig. 1B, Fig. S1). Given the abundance of immune cells within tumor stromal cells, we sought to ascertain the expression and distribution characteristics of PKN2 in this context. To this end, we consulted the BioGPS database (http://biogps.org/#goto=genereport_id=5586), which revealed that PKN2 is highly expressed in myeloid cells, particularly in neutrophils (Fig. 1C). Immunofluorescence staining results showed that PKN2 was expressed on MDSCs in esophageal cancer tissues (Fig. 1D). Similar results were observed in normal murine tissues, indicating that PKN2 was highly expressed in MDSCs, as opposed to macrophages, B cells, monocytes, or plasma cells (Fig. 1E). Furthermore, the expression of PKN2 was examined in ten paired clinical samples, including PBMCs and tumor tissues. The results demonstrated that, in comparison to PBMCs, PKN2 expression was elevated in myeloid cells, M-MDSCs, and polymorphonuclear myeloid derived suppressor cells (PMN-MDSCs) of tumor tissues (Fig. 1F). Furthermore, we examined the correlation between PKN2 expression in tumor tissues and the clinical characteristics of ESCC patients. Our findings indicated a significant correlation between PKN2 expression in PMN-MDSCs of tumor tissues and tumor stage. However, no correlation was observed between PKN2 expression and sex, tumor location, tumor differentiation, or local lymphatic metastasis (Table 1).

**Table 1:** Correlation analysis between clinical parameters of ESCC patients and PKN2 expression in PMN-MDSCs

Clinical features	*N* (Total *N* = 40)	PKN2 expression		*P* value
		Low (*N*, %)	High (*N*, %)	
Sex				
Male Female	28 12	12 (30.0) 7 (17.5)	16 (40.0) 5 (12.5)	0.369
Tumor location				
Upper Middle Lower	9 20 11	6 (15.0) 9 (22.5) 4 (10.0)	3 (7.5) 11(27.5) 7 (17.5)	0.382
Tumor stage				
I-II III-IV	29 11	17 (42.5) 2 (5.0)	12 (30.0) 9 (22.5)	**0.022**
Tumor differentiation				
Poor Moderate Well	15 17 8	8 (20.0) 7 (17.5) 4 (10.0)	7 (17.5) 10 (25.0) 4 (10.0)	0.780
Local lymphatic metastasis				
Yes No	23 17	9 (22.5) 10 (25.0)	14 (35.0) 7 (17.5)	0.218

*Note*: According to the relative expression of PKN2 in PMN-MDSCs of tumor tissues, 40 ESCC patients were divided into the PKN2 high-expression group (*N* = 21) and the PKN2 low-expression group (*N* = 19). The χ^2^ text was used in statistical analysis

**Fig. 2:**
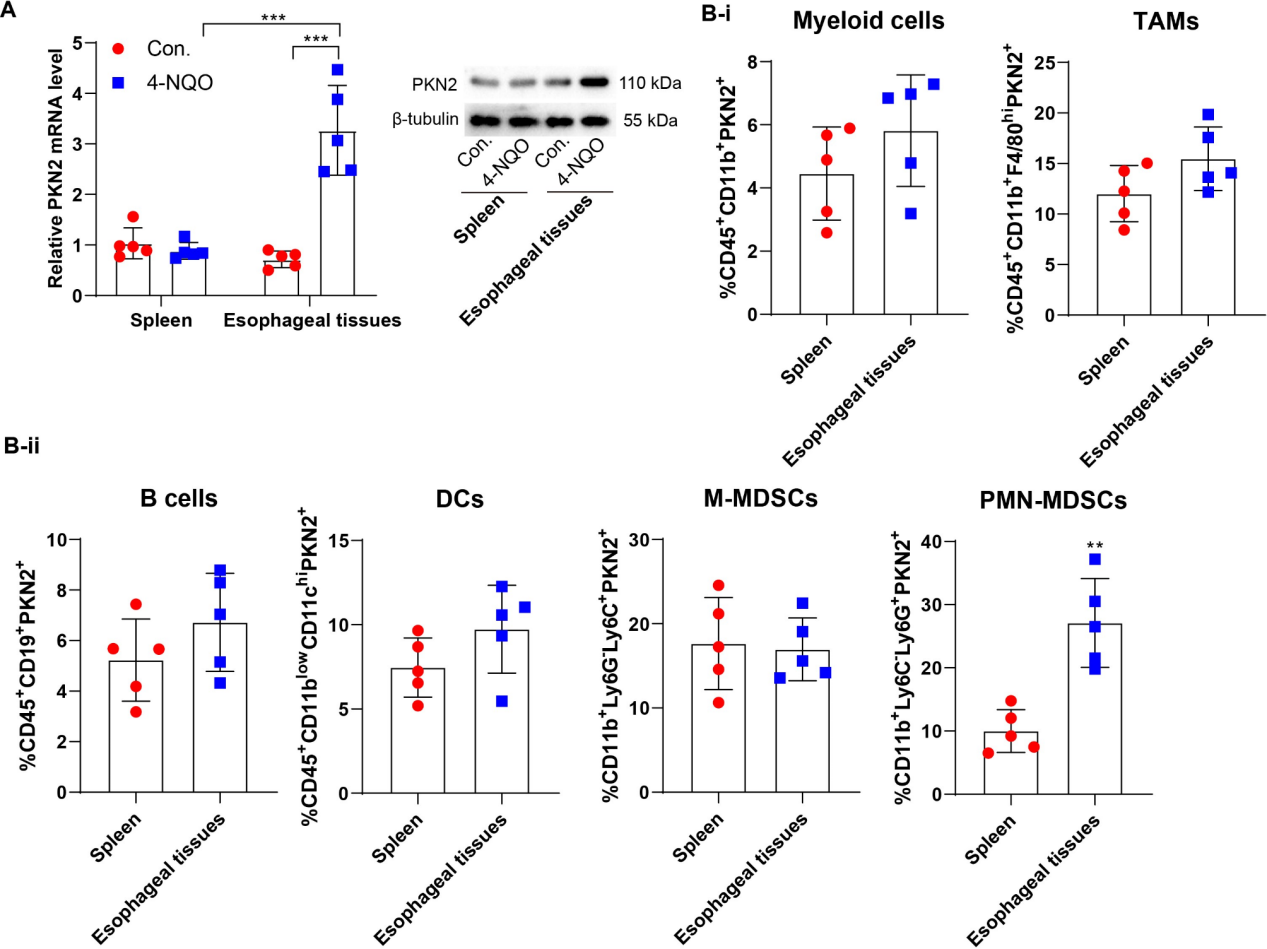
PKN2 is highly expressed in PMN-MDSCs of EC mouse tumor tissues. (**A**) Spontaneous carcinogenesis was induced by 4-NQO. Mouse spleen tissues and esophageal tissues were collected. The expression of PKN2 mRNA and protein levels were determined using qRT-PCR and Western blotting (*N* = 5). Statistical analysis: two-way ANOVA with Tukey’s post-hoc test. ***P < 0.001. (**B**) The percentage of myeloid cells, TAMs, B cells, DCs, M-MDSCs, and PMN-MDSCs in spleen and esophageal tissues of EC mice was detected using flow cytometry (*N* = 5). Statistical analysis: two tailed paired student’s t-test. **P < 0.01 vs. spleen

The expression of PKN2 was observed in mouse models of EC induced by 4-NQO. In mouse esophageal tissues, PKN2 expression was elevated in 4-NQO-induced EC mice; however, in mouse spleen tissues, PKN2 expression was not significantly elevated (Fig. 2A). In EC mouse models, the results of the flow cytometry analysis indicated that there was no statistically significant difference in PKN2 expression levels in myeloid cells, M-MDSCs, tumor associated macrophages (TAMs), B cells, and dendritic cells (DCs) isolated from spleen and tumor tissues. However, a notable increase in PKN2 expression was observed in tumor-derived PMN-MDSCs compared to their spleen-derived counterparts (Fig. 2B). These findings suggest that the increased expression of PKN2 in PMN-MDSCs may be associated with the progression of ESCC.

### PKN2 contributes to the immunosuppressive activity of PMN-MDSCs *in vitro*

**Fig. 3:**
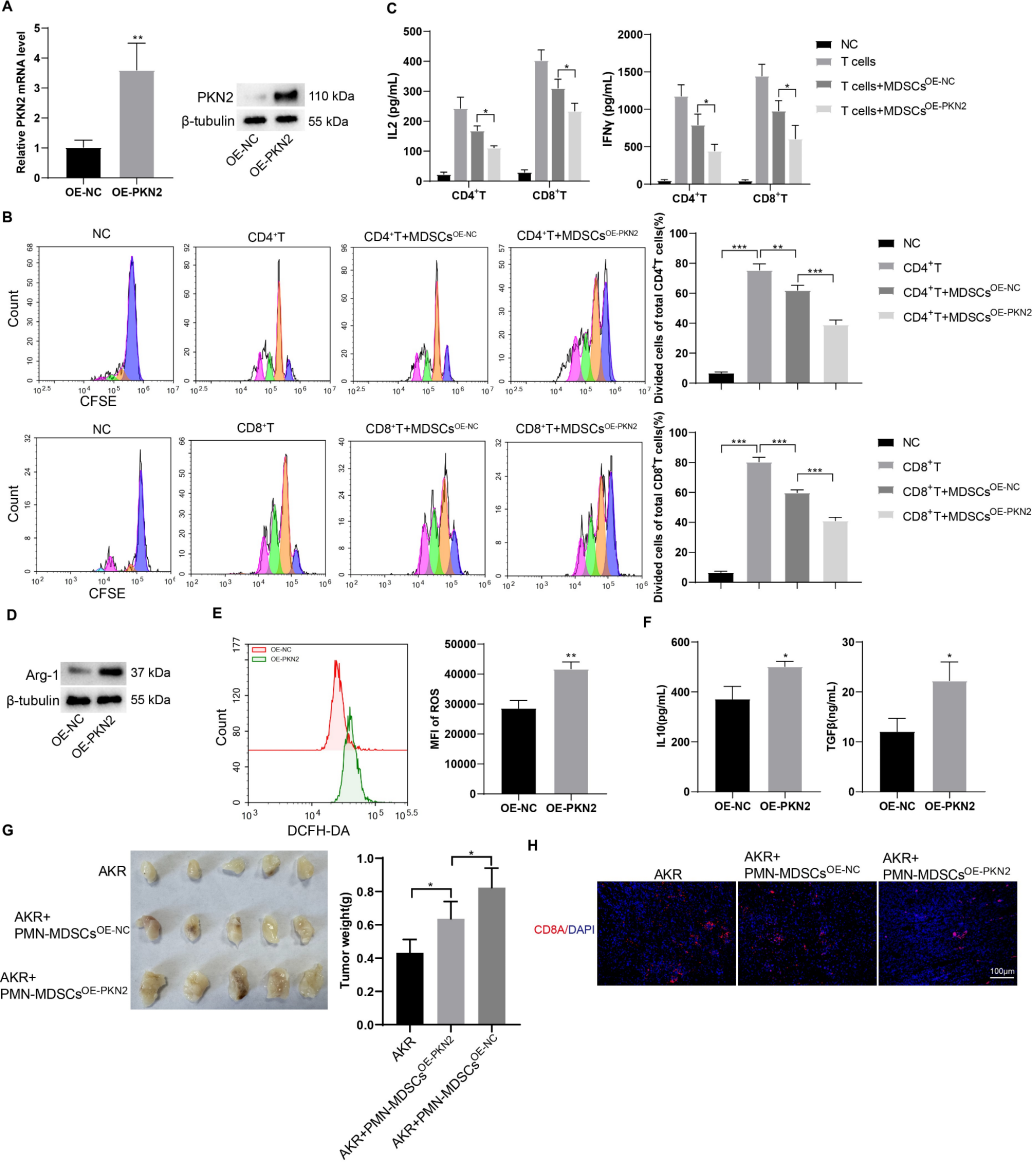
PKN2 contributes to the immunosuppressive activity of PMN-MDSCs *in vitro.* (**A**) PMN-MDSCs were isolated from EC mouse spleen tissues. Following transfection of the PKN2 overexpression (OE) lentivirus, mRNA level and protein level of PKN2 were detected using qRT-PCR and Western blotting, respectively (*N* = 3). Statistical analysis: two tailed un-paired student’s t-test. **P < 0.01 vs. OE-NC. (**B**) A co-culture system of PMN-MDSCs and CD4^+^/CD8^+^ T cells was established. The proliferation of T cells was detected by CFSE staining and flow cytometry (*N* = 3). Statistical analysis: one-way ANOVA with Tukey’s post-hoc test. **P < 0.01, ***P < 0.001. (**C**) Soluble IL-2 and IFN-γ levels in the supernatants of the co-culture system were determined by ELISA (*N* = 3). Statistical analysis: two-way ANOVA with Tukey’s post-hoc test. *P < 0.05. (**D**) Arg-1 expression was detected using Western blotting. (**E**) ROS generation was detected using the DCFH-DA probe and the fluorescence intensity was determined using flow cytometry (*N* = 3). Statistical analysis: two tailed un-paired student’s t-test. **P < 0.01 vs. OE-NC. (**F**) IL-10 and TGF-β levels in the supernatants of the co-culture system were determined by ELISA (*N* = 3). Statistical analysis: two tailed un-paired student’s t-test. *P < 0.05 vs. OE-NC. (**G**) Mouse esophageal cancer cells AKR were injected subcutaneously into C57/BL mice. One week later, PMN-MDSCs (infected with PKN2 overexpressing lentivirus and negative control vector, respectively) were injected intratumorally into the tumors (*N* = 5). The volume of subcutaneous tumors was measured and recorded every 3 days, and tumor tissue was collected by executing the mice on day 15. Statistical analysis: one-way ANOVA with Tukey’s post-hoc test. *P < 0.05. (**H**) Subcutaneous tumor tissues were subjected to immunofluorescence staining using CD8A (CD8^+^ T marker) antibody (red) and DAPI (blue)

Given that PKN2 expression was elevated in PMN-MDSCs derived from human and mouse EC tissues, we proceeded to investigate the impact of PKN2 on the activity and function of PMN-MDSCs. PMN-MDSCs were isolated from EC mouse spleen tissues and identified by flow cytometry (Fig. S2). Following transfection of the PKN2 overexpression lentivirus, a marked elevation in PKN2 was observed in mouse PMN-MDSCs (Fig. 3A). To observe the effects of PKN2 on the activity and function of PMN-MDSCs, a co-culture system of PMN-MDSCs and CD4^+^/CD8^+^ T cells was established. The overexpression of PKN2 markedly inhibited T cell proliferation in both groups during the co-culture system (Fig. 3B). Moreover, PMN-MDSCs that overexpress PKN2 exhibited a significant reduction in the secretion of soluble IL-2 and IFN-γ by CD4^+^/CD8^+^ T cells (Fig. 3C). The impact of PKN2 on the secretion of immunosuppressive molecules by PMN-MDSCs was subsequently examined. The results demonstrated that PKN2 overexpression augmented the expression of arginase-1 (Arg-1), ROS generation, and the secretion levels of IL-10 and TGF-β (Fig. 3D-F). PKN2 overexpression also enhanced the immunosuppressive activity of PMN-MDSCs *in vivo.* PMN-MDSCs increased tumor growth, and PKN2 overexpression further increased the promotion of PMN-MDSCs on tumor growth (Fig. 3G). PMN-MDSCs decreased the number of CD8^+^ T cells in tumor tissues, and PKN2 overexpression further increased the number of CD8^+^ T cells (Fig. 3H). These findings suggest that PKN2 plays a role in the immunosuppressive activity of PMN-MDSCs.

### PKN2-highly expressed PMN-MDSCs inhibit cytotoxic T lymphocytes (CTL) killing ability to promote ESCC organoid activity

**Fig. 4:**
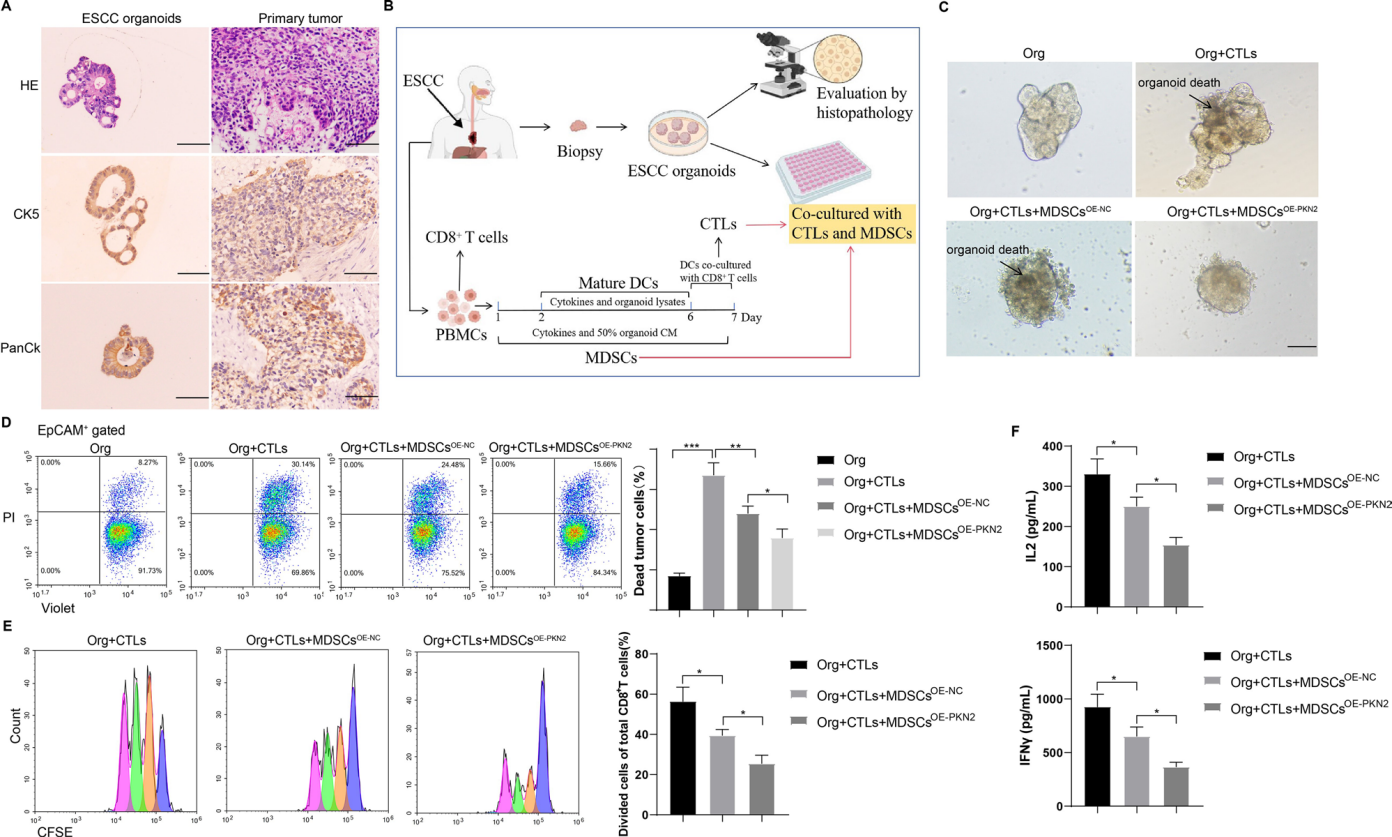
PKN2-highly expressed PMN-MDSCs inhibit CTL killing ability to promote ESCC organoid activity. (**A**) HE staining and immunohistochemistry (anti-PanCK and anti-CK5) of ESCC organoids and their primary tumor tissues. Scale bar = 100 μm. (**B**) The methodology illustrated the co-culture of ESCC organoids and CTLs. (**C**) Bright field results of the co-culture system. Scale bar = 100 μm. (**D**) Cell death was observed by flow cytometry using PI and Violet antibodies (*N* = 3). Statistical analysis: one-way ANOVA with Tukey’s post-hoc test. *P < 0.05, **P < 0.01, ***P < 0.001. (**E**) The proliferation of T cells was detected by CFSE staining and flow cytometry (*N* = 3). Statistical analysis: one-way ANOVA with Tukey’s post-hoc test. *P < 0.05. (**F**) Soluble IL-2 and IFN-γ levels in the supernatants of the co-culture system were determined by ELISA (*N* = 3). Statistical analysis: one-way ANOVA with Tukey’s post-hoc test. *P < 0.05

The co-culture of patient-derived organoids (PDOs) and autologous immune cells has been proposed as a valuable ex vivo surrogate model of the in vivo tumor-immune environment. To identify the immune interactions between PDOs and autologous immune cells, we initially established ESCC organoids from patient tumor tissues. A histological examination employing HE staining and immunohistochemistry (Pan-CK labels tumor cells; CK5 is a squamous cell carcinoma marker) revealed that ESCC organoids and tumor tissues were homogeneous (Fig. 4A). The co-culture of ESCC organoids and CTLs was conducted in accordance with the methodology illustrated in Fig. 4B. Prior to the addition of PMN-MDSCs, the co-culture system displayed indications of organoid death. However, following the introduction of PMN-MDSCs that highly expressed PKN2, ESCC organoids exhibited robust growth and minimal organoid death (Fig. 4C). Moreover, the introduction of PMN-MDSCs with high PKN2 expression resulted in an increase in the number of viable cells within the ESCC organoids (PI^−^Violet^+^) while simultaneously reducing the number of dead cells (PI^+^Violet^+^) (Fig. 4D). Additionally, the proliferation of CD8^+^ T cells was observed to be diminished (Fig. 4E), and the secretion of soluble IL-2 and IFN-γ by CD8^+^ T cells was also reduced (Fig. 4F). These findings suggest that the presence of PMN-MDSCs with high PKN2 expression may impede the ability of CTLs to eliminate ESCC organoids, thereby facilitating their proliferation.

### PKN2 promotes fatty acid oxidation (FAO) in PMN-MDSCs via CPT1B

**Fig. 5:**
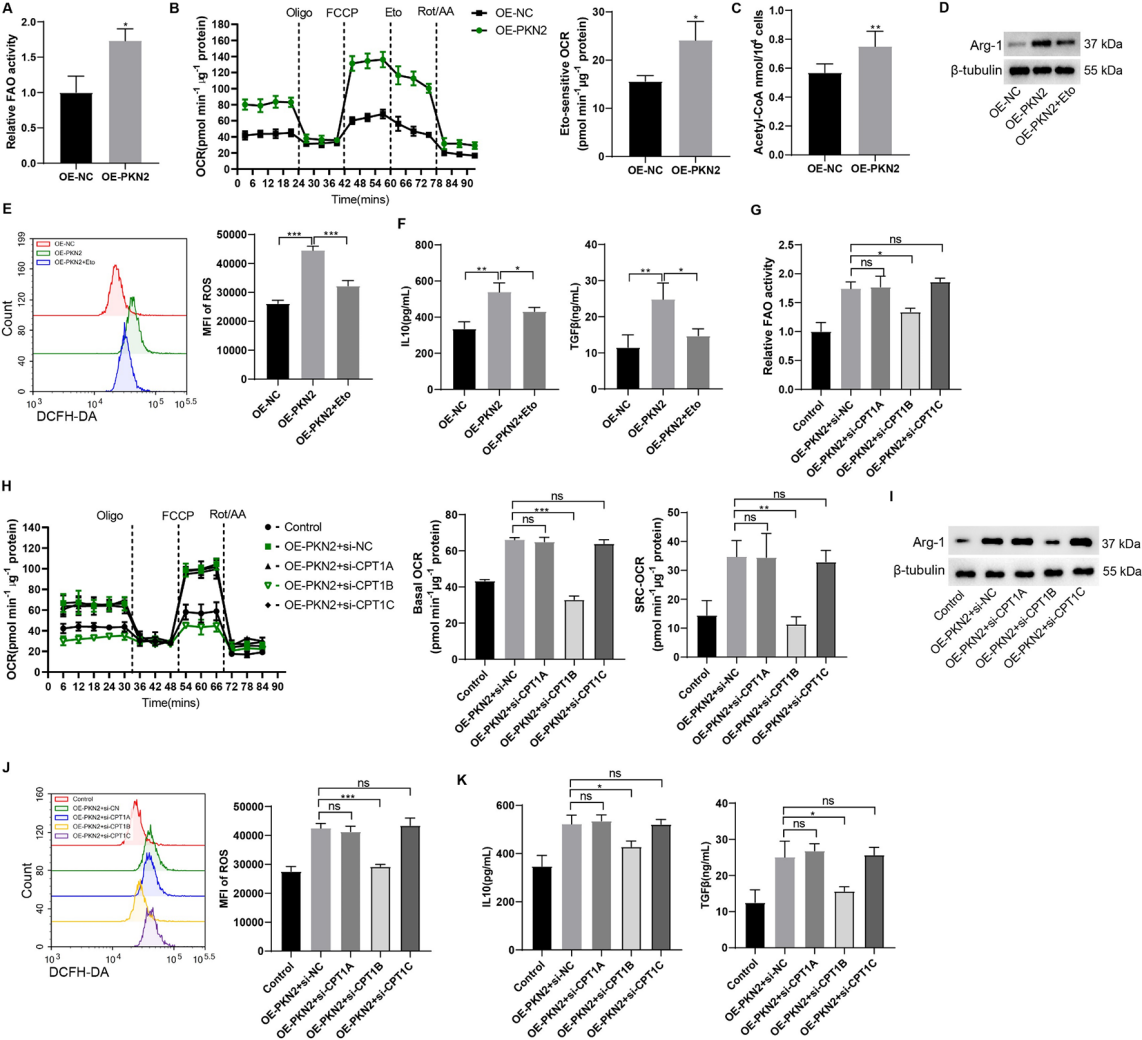
PKN2 promotes fatty acid oxidation in PMN-MDSCs. (**A**) PMN-MDSCs were isolated from EC mouse spleen tissues and subsequently transfected with the PKN2 overexpression lentivirus. The modulation of FAO by PKN2 in PMN-MDSCs was detected using the Fatty Acid Oxidation Assay Kit (*N* = 3). Statistical analysis: two tailed un-paired student’s t-test. *P < 0.05 vs. OE-NC. (**B**) Following a 24-hour incubation period, the OCR was detected using Oligomycin (4 µM), FCCP (1.6 µM), rotenone (0.5 µM) + antimycin A (0.5 µM), and etomoxir (18 µM, an FAO inhibitor) (*N* = 3). Statistical analysis: two tailed un-paired student’s t-test. *P < 0.05 vs. OE-NC. (**C**) The effect of PKN2 on acetyl-CoA levels in PMN-MDSCs (*N* = 3). Statistical analysis: two tailed un-paired student’s t-test. **P < 0.01 vs. OE-NC. (**D**) Arg-1 expression was detected using Western blotting. (**E**) ROS generation was detected using the DCFH-DA probe and the fluorescence intensity was determined using flow cytometry (*N* = 3). Statistical analysis: one-way ANOVA with Tukey’s post-hoc test. ***P < 0.001. (**F**) IL-10 and TGF-β levels in the supernatants were determined by ELISA (*N* = 3). Statistical analysis: one-way ANOVA with Tukey’s post-hoc test. *P < 0.05. **P < 0.01. (**G**) PMN-MDSCs were isolated from EC mouse spleen tissues and subsequently transfected with the PKN2 overexpression lentivirus and CPT1A/B/C siRNAs. The modulation of FAO was detected using the Fatty Acid Oxidation Assay Kit (*N* = 3). Statistical analysis: one-way ANOVA with Tukey’s post-hoc test. *P < 0.05. (**H**) Following a 24-hour incubation period, the OCR was detected (*N* = 3). Statistical analysis: one-way ANOVA with Tukey’s post-hoc test. **P < 0.01. ***P < 0.001. (**I**) Arg-1 expression was detected using Western blotting. (**J**) ROS generation was detected using the DCFH-DA probe and the fluorescence intensity was determined using flow cytometry (*N* = 3). Statistical analysis: one-way ANOVA with Tukey’s post-hoc test. ***P < 0.001. (**K**) IL-10 and TGF-β levels in the supernatants were determined by ELISA (*N* = 3). Statistical analysis: one-way ANOVA with Tukey’s post-hoc test. *P < 0.05

PMN-MDSCs were isolated from EC mouse spleen tissues and subsequently transfected with the PKN2 overexpression lentivirus. We detected the modulation of FAO by PKN2 in PMN-MDSCs and the results of the assay showed that the modulation of FAO was increased after PKN2 overexpression (Fig. 5A). Following a 24-hour incubation period, the OCR data demonstrated that PKN2 overexpression enhanced FAO-dependent OCR in PMN-MDSCs (Fig. 5B). We examined the effect of PKN2 on acetyl-CoA levels in PMN-MDSCs, and the results showed that acetyl-CoA levels were increased in PMN-MDSCs after PKN2 overexpression (Fig. 5C). Subsequently, the FAO inhibitor etomoxir (Eto) was introduced to the culture medium. The introduction of etomoxir resulted in a notable reduction in PKN2-induced Arg-1 expression (Fig. 5D), ROS generation (Fig. 5E), and IL-10 and TGF-β secretion (Fig. 5F), indicating that FAO plays a crucial role in mediating the enhanced activity of PKN2-treated PMN-MDSCs. Carnitine palmitoyltransferase 1 (CPT1) represents the rate-limiting step of FAO, facilitating the translocation of long-chain fatty acids to mitochondria. Three distinct isoforms of CPT1 have been identified: CPT1A, CPT1B, and CPT1C. To validate the crucial role of FAO in the activity of PKN2-increased PMN-MDSCs, we silenced CPT1A, CPT1B, and CPT1C, respectively, by using small interfering RNAs (siRNAs) and confirmed the transfection efficiency (Fig. S3). The silencing of CPT1B was observed to significantly reduce the up-regulation of FAO modulation (Fig. 5G) and OCR (Fig. 5H) induced by overexpression of PKN2 in PMN-MDSCs. In contrast, silencing CPT1A or CPT1C had no significant effect on the OCR of PMN-MDSCs overexpressing PKN2 (Fig. 5H). In accordance with the outcomes of the OCR assay, silencing CPT1B led to a notable reduction in PKN2-induced Arg-1 expression (Fig. 5I), ROS generation (Fig. 5J), and IL-10 and TGF-β secretion (Fig. 5K), whereas silencing CPT1A or CPT1C did not elicit comparable outcomes. The results serve to further confirm that PKN2 enhances the activity of PMN-MDSCs by promoting FAO. Furthermore, these results suggest that PKN2 induces CPT1B-dependent FAO in PMN-MDSCs.

### PKN2 promotes CPT1B transcription by mediating STAT3 phosphorylation

**Fig. 6:**
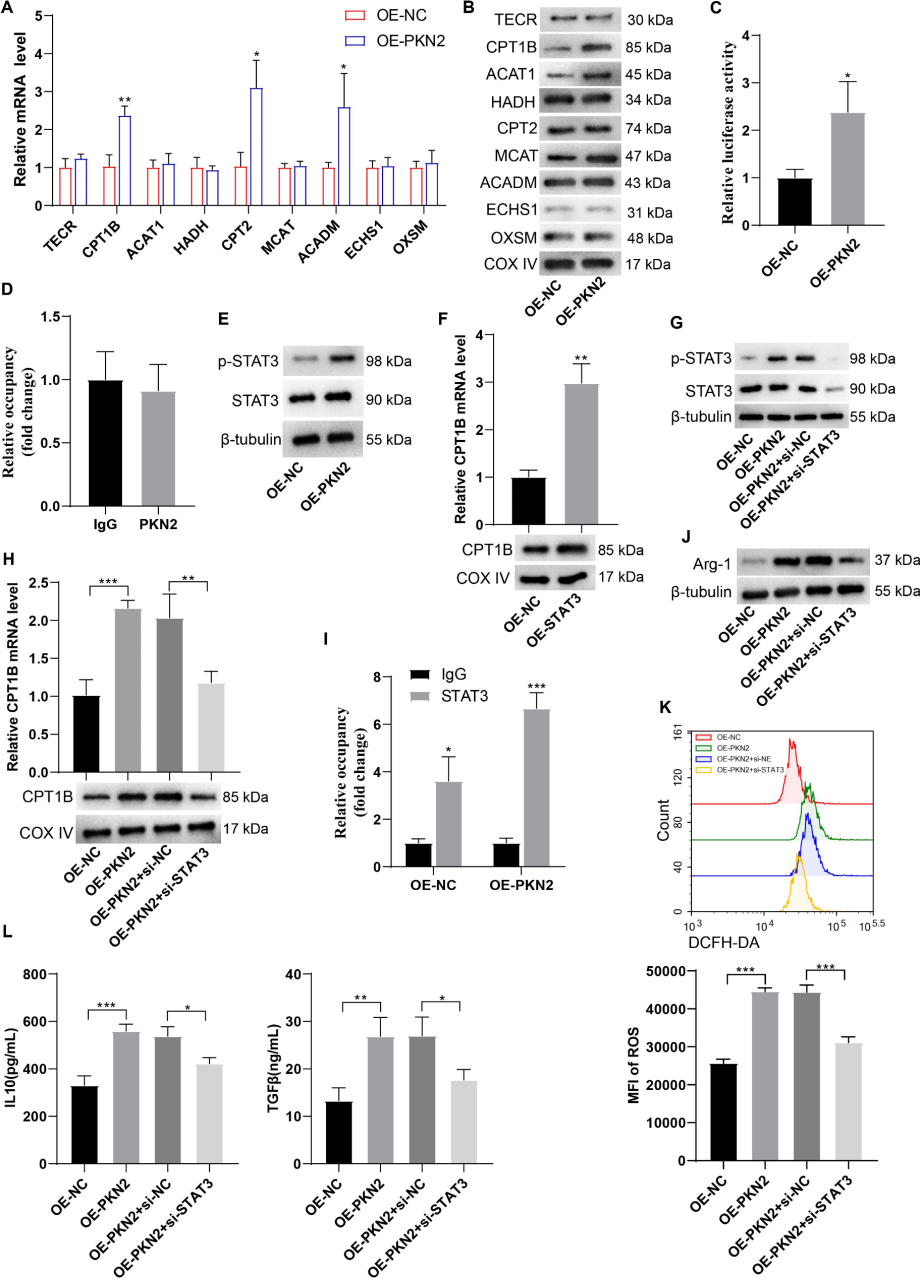
PKN2 promotes CPT1B transcription through STAT3. (**A**) PMN-MDSCs were isolated from EC mouse spleen tissues and subsequently transfected with the PKN2 overexpression lentivirus. Expressions of FAO-related genes, including TECR, CPT1B, ACAT1, HADH, CPT2, MCAT, ACADM, ECHS1, and OXSM, were detected using qRT-PCR (*N* = 3). Statistical analysis: one-way ANOVA with Tukey’s post-hoc test. *P < 0.05, **P < 0.01 vs. OE-NC. (**B**) Protein expressions of FAO-related genes were detected using Western blotting. (**C**) The promotion of CPT1B transcription by PKN2 was confirmed using the dual luciferase reporter gene assay (*N* = 3). Statistical analysis: two tailed un-paired student’s t-test. *P < 0.05 vs. OE-NC. (**D**) The binding between PKN2 and CPT1B promoter was detected using ChIP-qPCR (*N* = 3). Statistical analysis: two tailed un-paired student’s t-test. (**E**) Total STAT3 and p-STAT3 levels in PKN2-overexpressing PMN-MDSCs were detected using Western blotting. (**F**) The overexpression of STAT3 in PMN-MDSCs led to an increase in CPT1B expression (*N* = 3). Statistical analysis: two tailed un-paired student’s t-test. **P < 0.01 vs. OE-NC. (**G**) PMN-MDSCs were transfected with PKN2 overexpression lentivirus or together with STAT3 siRNAs. Total STAT3 and p-STAT3 levels were detected using Western blotting. (**H**) mRNA level and protein level of CPT1B were detected using qRT-PCR and Western blotting, respectively (*N* = 3). Statistical analysis: one-way ANOVA with Tukey’s post-hoc test. **P < 0.01, ***P < 0.001. (**I**) The binding between STAT3 and CPT1B promoter was detected using ChIP-qPCR (*N* = 3). Statistical analysis: two-way ANOVA with Tukey’s post-hoc test. *P < 0.05, ***P < 0.001. (**J**) Arg-1 expression was detected using Western blotting. (**K**) ROS generation was detected using the DCFH-DA probe and the fluorescence intensity was determined using flow cytometry (*N* = 3). Statistical analysis: one-way ANOVA with Tukey’s post-hoc test. ***P < 0.001. (**L**) IL-10 and TGF-β levels in the supernatants were determined by ELISA (*N* = 3). Statistical analysis: one-way ANOVA with Tukey’s post-hoc test. *P < 0.05, **P < 0.01, ***P < 0.001

To elucidate the downstream target genes regulated by PKN2, we examined the effect of PKN2 on the expression of FAO-related genes, including TECR, CPT1B, ACAT1, HADH, CPT2, MCAT, ACADM, ECHS1, and OXSM. The results demonstrated that the overexpression of PKN2 markedly elevated the expression of CPT1B, CPT2, and ACADM mRNAs (Fig. 6A). Western blotting revealed that CPT1B protein was significantly increased in PKN2-overexpressing PMN-MDSCs, and PKN2 also enhanced ACAT1 and MCAT protein expression (Fig. 6B). Given that PKN2 promoted both CPT1B mRNA and protein expression and the critical role of CPT1B in FAO, we postulated that PKN2 might promote FAO in PMN-MDSCs by regulating CPT1B. A dual luciferase reporter gene assay demonstrated that PKN2 enhances CPT1B transcription, as evidenced by the increased luciferase activity observed in HEK293 cells upon PKN2 overexpression (Fig. 6C). The ChIP-qPCR assay demonstrated that PKN2 did not bind to the CPT1B promoter, indicating that PKN2 does not directly promote CPT1B transcription but rather regulates CPT1B transcription through other molecules (Fig. 6D). STAT3 is one of the key molecules regulating the expansion and activation of tumor MDSCs and can modulate the immunosuppressive activity of MDSCs through multiple mechanisms ((Bitsch et al. 2022). STAT3 has been shown to promote CPT1B transcription (Wang et al. 2018). Phosphorylation modification is a critical part of STAT3 activation (Dong et al. 2021). Given PKN2 kinase activity, we hypothesized that PKN2 promotes CPT1B transcription by mediating STAT3 phosphorylation. It was observed that PKN2 increased the phosphorylation level of Signal transducer and activator of transcription 3 (STAT3) serine 727 (S727) in PMN-MDSCs (Fig. 6E).

STAT3 plays a pivotal role in the expansion and activation of tumor-associated myeloid-derived suppressor cells (MDSCs). It exerts its immunosuppressive effects on MDSCs through a multitude of mechanisms (Bitsch et al. 2022). A previous study has confirmed that STAT3 promotes CPT1B transcription (Wang et al. 2018). In addition, phosphorylation modification is a crucial part of STAT3 activation process (Dong et al. 2021). Accordingly, we postulated that PKN2 facilitates CPT1B transcription by orchestrating STAT3 phosphorylation. The subsequent experiments demonstrated that the overexpression of STAT3 in PMN-MDSCs led to an increase in CPT1B expression (Fig. 6F), whereas the silencing of STAT3 resulted in a reduction in the promotion of p-STAT3 (Fig. 6G) and CPT1B expression (Fig. 6H) by PKN2. The ChIP-qPCR assay demonstrated that PKN2 facilitated the binding of STAT3 to the CPT1B promoter (Fig. 6I). Furthermore, the silencing of STAT3 resulted in a notable reduction in PKN2-induced Arg-1 expression (Fig. 6J), ROS generation (Fig. 6K), and IL-10 and TGF-β secretion (Fig. 6L). Similarly, the administration of STAT3 inhibitor Napabucasin inhibits the promotion of CPT1B expression and MDSC immunosuppressive capacity by PKN2 (Fig. S4). In conclusion, the data collectively suggest that PKN2 facilitates CPT1B transcription by modulating STAT3 phosphorylation.

## Discussion

The standard treatment modalities for EC encompass surgical, chemotherapeutic, and radiotherapeutic approaches. However, due to the absence of overt early symptoms, a considerable proportion of patients present with advanced disease at the time of diagnosis, which subsequently constrains the efficacy of treatment (Zhang et al. 2024). Furthermore, in cases where surgical resection is extensive, patients who have experienced complications and elderly patients frequently lack the physiological capacity to withstand the procedure. The median survival time for patients with advanced EC is relatively brief, and the five-year survival rate is low. Immunotherapy has become a fundamental component of EC treatment regimens, resulting in improved survival outcomes for patients. However, not all patients with EC respond to immunotherapy. The presence of MDSCs within the tumor microenvironment has been demonstrated to result in immunosuppression and the failure of immunotherapy. Our findings indicated that PKN2 expression was increased in PMN-MDSCs derived from human and mouse EC tissues. From a mechanistic standpoint, PKN2 plays a role in the augmentation of PMN-MDSC immunosuppressive activity by facilitating STAT3 phosphorylation and CPT1B transcription, which in turn leads to elevated CPT1B-mediated FAO in PMN-MDSCs. The targeted inhibition of PKN2 is anticipated to enhance the immunotherapeutic efficacy of EC patients.

PKN is widely distributed in tissues of various species, from lower organisms to mammals, and its functions are diverse and context-specific. PKN2 (Protein kinase N2), also known as Pak-2, PRK2, PRKCL2, or STK7, is a PKC-related serine/threonine protein kinase and a Rho/Rac effector protein that plays a role in specific signaling responses in cells (Al-Sha’er et al. 2023). It performs regulatory functions in cell cycle progression, cytoskeleton assembly, cell migration, cell adhesion, tumor cell invasion, and transcriptional activation signaling (Yang et al. 2017). In tumors, PKN2 regulates the velocity and trajectory of epithelial bladder cells during cell migration and tumor cell invasion (Lachmann et al. 2011). In the context of tumor immunomodulation, PKN2 has been observed to inhibit the polarization of M2-type TAMs in colorectal cancer cells (Cheng et al. 2018). Moreover, the expression of PKN2 in colorectal cancer cells has been associated with a favorable prognosis. In a mouse xenograft model, PKN2 has demonstrated the capacity to inhibit tumor growth and suppress M2-type polarization, both in vitro and in vivo (Cheng et al. 2018). Furthermore, we investigated the correlation between clinical parameters of ESCC patients and PKN2 expression in ESCC tumor tissues. Our findings revealed a significant correlation between PKN2 expression in PMN-MDSCs of tumor tissues and tumor stage. However, no correlation was observed between PKN2 expression and sex, tumor location, tumor differentiation, or local lymphatic metastasis. It is important to note that the sample size of patients included in this study was relatively small. Therefore, further investigation is necessary to confirm the observed correlation between PKN2 and the clinical characteristics of esophageal cancer patients by expanding the sample size.

It is notable that the role of PKN2 in tumor immunomodulation may be associated with the activation of additional immune cell types and inflammatory vesicles, as well as with cellular and molecular mechanisms that play a pivotal role in the tumor microenvironment (Huang et al. 2023). PKN2 may regulate tumor growth and metastasis by influencing the activity of immune cells and the composition of the tumor microenvironment (Murray et al. 2022). This study concentrated on the regulation of tumor-infiltrating PMN-MDSCs by PKN2 and corroborated the elevated expression of PKN2 in MDSCs, particularly PMN-MDSCs, through the utilization of the GEO database, clinical specimens from ESCC patients, and artificially induced EC mouse models. Subsequent studies indicated that PKN2 may enhance FAO by indirectly regulating CPT1B transcription, which ultimately enhances the immunosuppressive activity of PMN-MDSCs. In light of these findings, PKN2 may represent a promising therapeutic target in tumor immunotherapy. The targeted inhibition of its signaling pathway may represent a novel strategy for tumor treatment.

Metabolic reprogramming is a vital process for tumor cells, enabling them to fulfill the bioenergetic and biosynthetic requirements of rapid cellular proliferation and adaptation to the tumor microenvironment (Tang et al. 2024; Wang et al. 2024a, b). FAO plays a crucial role in the metabolic process of MDSCs. MDSCs enhance lipid uptake through the upregulation of the scavenger receptor CD36, relying on FAO as a primary source of energy (Veglia et al. 2021). The metabolic reprogramming of FAO is of paramount importance for the differentiation and functionality of MDSCs. Inhibition of FAO has the potential to affect the immunosuppressive capacity of MDSCs, thereby delaying tumor growth and improving the efficacy of chemotherapy and immunotherapy (Veglia et al. 2021). CPT1B is a pivotal enzyme in the FAO process, which functions to catalyze the binding of long-chain fatty acids to coenzyme A, facilitating the entry of fatty acids into the mitochondria for β-oxidation and energy production (Schlaepfer and Joshi 2020). It has been demonstrated that CPT1B deficiency results in enhanced cardiac dysfunction and hypertrophy, particularly in the context of pressure overload conditions (Li et al. 2023). Furthermore, the expression level of CPT1B has been demonstrated to exert a significant influence on intramyocardial fatty acid metabolism (Angelini et al. 2021). A study of Mstn gene disruption demonstrated that the downregulation of Mstn promoted β-oxidative metabolism of intramyocardial fatty acids by upregulating Cpt1b expression (Guo et al. 2022). These findings suggest that CPT1B plays a crucial role in the regulation of fatty acid metabolism and energy homeostasis. In the current study, the silencing of CPT1B was observed to significantly reduce the up-regulation of OCR induced by overexpression of PKN2 in PMN-MDSCs. In contrast, silencing CPT1A or CPT1C had no significant effect on the OCR of PMN-MDSCs overexpressing PKN2. In accordance with the outcomes of the OCR assay, silencing CPT1B led to a notable reduction in PKN2-induced Arg-1 expression, ROS generation, and IL-10 and TGF-β secretion, whereas silencing CPT1A or CPT1C did not elicit comparable outcomes. Therefore, CPT1B was identified as the major regulator of FAO in EC infiltrated PMN-MDSCs.

It is noteworthy that fatty acid transporter protein 2 (FATP2) serves as a regulator of immunosuppressive function in MDSCs (Sanchez-Pino et al. 2021). FATP2 is responsible for arachidonate utilization and prostaglandin 2 synthesis, whereas inhibition of FATP2 expression abrogates immunosuppressive capacity in PMN-MDSCs and enhances the efficacy of cancer immunotherapy (Veglia et al. 2021). Despite the absence of direct mention in previous studies, evidence suggests the existence of a regulatory relationship between CPT1B and FATP2. However, with regard to their respective functions, both CPT1B and FATP2 are involved in fatty acid metabolism, although they have different points of action in this process. CPT1B primarily acts in the β-oxidation phase of fatty acids, whereas FATP2 is involved in fatty acid uptake and transport. Although they perform disparate functions in fatty acid metabolism, their activities are interrelated, collectively maintaining the lipid metabolic homeostasis of the cell. Given that both can affect the immunosuppressive capacity of MDSC, it is plausible that the two may collaborate with each other in metabolic pathways. However, the specific regulatory mechanisms involved may require further investigation to be fully elucidated.

In conclusion, this study examined the expression level of PKN2 in PMN-MDSCs derived from human and mouse EC tissues and found that PKN2 plays a critical role in promoting the immunosuppressive function of PMN-MDSCs in EC. This mechanism is associated with the STAT3-mediated transcriptional elevation of CPT1B. Moreover, our study utilized a co-culture system comprising ESCC organoids and T cells, thereby emphasizing the utility of the PDO co-culture system as a valuable ex vivo surrogate model of the in vivo tumor-immune environment. Our findings suggest that PKN2 may represent a promising therapeutic target for improving the efficacy of immunotherapy in EC.

## Electronic supplementary material

Below is the link to the electronic supplementary material.

Supplementary Material 1

## Data Availability

No datasets were generated or analysed during the current study.

## References

[CR27] Al-Sha’er MA, Basheer HA, Taha MO. Discovery of new PKN2 inhibitory chemotypes via QSAR-guided selection of docking-based pharmacophores[J]. Mol Divers. 2023;27(1):443–62. 35507210 10.1007/s11030-022-10434-4

[CR36] Angelini A, Saha PK, Jain A, et al. PHDs/CPT1B/VDAC1 axis regulates long-chain fatty acid oxidation in cardiomyocytes[J]. Cell Rep. 2021;37(1):109767. 34610308 10.1016/j.celrep.2021.109767 PMC8658754

[CR23] Bitsch R, Kurzay A, Özbay Kurt F et al. STAT3 inhibitor Napabucasin abrogates MDSC immunosuppressive capacity and prolongs survival of melanoma-bearing mice[J]. J Immunother Cancer, 2022, 10(3). 10.1136/jitc-2021-004384 PMC8932276 35301236

[CR19] Cattaneo CM, Dijkstra KK, Fanchi LF, et al. Tumor organoid-T-cell coculture systems[J]. Nat Protoc. 2020;15(1):15–39. 31853056 10.1038/s41596-019-0232-9 PMC7610702

[CR18] Chen X, Zhang H, Fang Z, et al. Adipocytes promote metastasis of breast cancer by attenuating the FOXO1 effects and regulating copper homeostasis[J]. Cancer Cell Int. 2024;24(1):284. 39135158 10.1186/s12935-024-03433-y PMC11320833

[CR17] Cheng Y, Zhu Y, Xu J, et al. PKN2 in colon cancer cells inhibits M2 phenotype polarization of tumor-associated macrophages via regulating DUSP6-Erk1/2 pathway[J]. Mol Cancer. 2018;17(1):13. 29368606 10.1186/s12943-017-0747-z PMC5784528

[CR1] Deboever N, Jones CM, Yamashita K, et al. Advances in diagnosis and management of cancer of the esophagus[J]. BMJ. 2024;385:e074962. 38830686 10.1136/bmj-2023-074962

[CR21] Dijkstra KK, Cattaneo CM, Weeber F, et al. Generation of Tumor-reactive T cells by co-culture of Peripheral Blood lymphocytes and Tumor Organoids[J]. Cell. 2018;174(6):1586–e15981512. 30100188 10.1016/j.cell.2018.07.009 PMC6558289

[CR25] Dong J, Cheng XD, Zhang WD, et al. Recent update on development of small-molecule STAT3 inhibitors for Cancer Therapy: from phosphorylation inhibition to protein Degradation[J]. J Med Chem. 2021;64(13):8884–915. 34170703 10.1021/acs.jmedchem.1c00629

[CR5] Gao J, Song Y, Kou X, et al. The effect of liver metastases on clinical efficacy of first-line programmed death-1 inhibitor plus chemotherapy in esophageal squamous cell carcinoma: a post hoc analysis of ASTRUM-007 and meta-analysis[J]. Cancer Med. 2024;13(10):e7203. 38769930 10.1002/cam4.7203 PMC11106639

[CR37] Guo Y, Yang R, Zhang Z, et al. [Mstn knockdown promotes intramuscular fatty acid metabolism by β oxidation via the up-regulation of Cpt1b][J]. Sheng Wu Gong Cheng Xue Bao. 2022;38(8):3076–89. 36002433 10.13345/j.cjb.210920

[CR2] Han B, Zheng R, Zeng H, et al. Cancer incidence and mortality in China, 2022[J]. J Natl Cancer Cent. 2024;4(1):47–53. 39036382 10.1016/j.jncc.2024.01.006 PMC11256708

[CR12] Hossain F, Al-Khami AA, Wyczechowska D, et al. Inhibition of fatty acid oxidation modulates immunosuppressive functions of myeloid-derived suppressor cells and enhances Cancer Therapies[J]. Cancer Immunol Res. 2015;3(11):1236–47. 26025381 10.1158/2326-6066.CIR-15-0036 PMC4636942

[CR30] Huang H, Weng Y, Tian W, et al. Molecular mechanisms of pyroptosis and its role in anti-tumor immunity[J]. Int J Biol Sci. 2023;19(13):4166–80. 37705746 10.7150/ijbs.86855 PMC10496503

[CR3] Kadono T, Yamamoto S, Kato K. Development of perioperative immune checkpoint inhibitor therapy for locally advanced esophageal squamous cell carcinoma[J]. Future Oncol, 2024:1–11. 10.1080/14796694.2024.2345043 PMC11497952 38861290

[CR8] Kapor S, Radojković M, Santibanez JF. Myeloid-derived suppressor cells: implication in myeloid malignancies and immunotherapy[J]. Acta Histochem. 2024;126(5–7):152183. 39029317 10.1016/j.acthis.2024.152183

[CR29] Lachmann S, Jevons A, De Rycker M, et al. Regulatory domain selectivity in the cell-type specific PKN-dependence of cell migration[J]. PLoS ONE. 2011;6(7):e21732. 21754995 10.1371/journal.pone.0021732 PMC3130767

[CR15] Li J, Dong W, Jiang Q, et al. LINC00668 cooperated with HuR dependent upregulation of PKN2 to facilitate gastric cancer metastasis[J]. Cancer Biol Ther. 2021;22(4):311–23. 33879018 10.1080/15384047.2021.1905138 PMC8183511

[CR35] Li X, Wu F, Günther S, et al. Inhibition of fatty acid oxidation enables heart regeneration in adult mice[J]. Nature. 2023;622(7983):619–26. 37758950 10.1038/s41586-023-06585-5 PMC10584682

[CR4] Li J, Xu C, Yuan S. A cost-effectiveness analysis of the combination of serplulimab with chemotherapy for advanced esophageal squamous cell carcinoma: insights from the ASTRUM-007 trial[J]. Cost Eff Resour Alloc. 2024;22(1):8. 38281053 10.1186/s12962-024-00516-5 PMC10821310

[CR16] Liu L, Chen Y, Lin X, et al. Upregulation of SNTB1 correlates with poor prognosis and promotes cell growth by negative regulating PKN2 in colorectal cancer[J]. Cancer Cell Int. 2021;21(1):547. 34663329 10.1186/s12935-021-02246-7 PMC8524951

[CR13] Marrocco V, Bogomolovas J, Ehler E, et al. PKC and PKN in heart disease[J]. J Mol Cell Cardiol. 2019;128:212–26. 30742812 10.1016/j.yjmcc.2019.01.029 PMC6408329

[CR11] Mohammadpour H, MacDonald CR, McCarthy PL, et al. β2-adrenergic receptor signaling regulates metabolic pathways critical to myeloid-derived suppressor cell function within the TME[J]. Cell Rep. 2021;37(4):109883. 34706232 10.1016/j.celrep.2021.109883 PMC8601406

[CR22] Monroy-Cárdenas M, Almarza C, Valenzuela-Hormazábal P et al. Identification of antioxidant methyl derivatives of Ortho-Carbonyl Hydroquinones that Reduce Caco-2 cell energetic metabolism and alpha-glucosidase Activity[J]. Int J Mol Sci, 2024, 25(15). 10.3390/ijms25158334 PMC11313435 39125904

[CR14] Murray ER, Menezes S, Henry JC, et al. Disruption of pancreatic stellate cell myofibroblast phenotype promotes pancreatic tumor invasion[J]. Cell Rep. 2022;38(4):110227. 35081338 10.1016/j.celrep.2021.110227 PMC8810397

[CR38] Sanchez-Pino MD, Dean MJ, Ochoa AC. Myeloid-derived suppressor cells (MDSC): when good intentions go awry[J]. Cell Immunol. 2021;362:104302. 33592540 10.1016/j.cellimm.2021.104302 PMC8054443

[CR34] Schlaepfer IR, Joshi M. CPT1A-mediated Fat Oxidation, mechanisms, and therapeutic Potential[J]. Endocrinology, 2020, 161(2). 10.1210/endocr/bqz046 31900483

[CR20] Sui Q, Liu D, Jiang W et al. Dickkopf 1 impairs the tumor response to PD-1 blockade by inactivating CD8 + T cells in deficient mismatch repair colorectal cancer[J]. J Immunother Cancer, 2021, 9(3). 10.1136/jitc-2020-001498 PMC8009229 33782107

[CR31] Tang Q, Wu S, Zhao B, et al. Reprogramming of glucose metabolism: the hallmark of malignant transformation and target for advanced diagnostics and treatments[J]. Biomed Pharmacother. 2024;178:117257. 39137648 10.1016/j.biopha.2024.117257

[CR33] Veglia F, Sanseviero E, Gabrilovich DI. Myeloid-derived suppressor cells in the era of increasing myeloid cell diversity[J]. Nat Rev Immunol. 2021;21(8):485–98. 33526920 10.1038/s41577-020-00490-y PMC7849958

[CR24] Wang T, Fahrmann JF, Lee H, et al. JAK/STAT3-Regulated fatty acid β-Oxidation is critical for breast Cancer Stem Cell Self-Renewal and Chemoresistance[J]. Cell Metab. 2018;27(1):136–e150135. 29249690 10.1016/j.cmet.2017.11.001 PMC5777338

[CR10] Wang R, Li B, Huang B et al. Gut Microbiota-Derived Butyrate Induces Epigenetic and Metabolic Reprogramming in Myeloid-Derived Suppressor Cells to Alleviate Primary Biliary Cholangitis[J]. Gastroenterology, 2024a. 10.1053/j.gastro.2024.05.014 38810839

[CR32] Wang H, Cui W, Yue S, et al. Malic enzymes in cancer: Regulatory mechanisms, functions, and therapeutic implications[J]. Redox Biol. 2024b;75:103273. 39142180 10.1016/j.redox.2024.103273 PMC11367648

[CR9] Wu T, Zhao Y, Wang H, et al. mTOR masters monocytic myeloid-derived suppressor cells in mice with allografts or tumors[J]. Sci Rep. 2016;6:20250. 26833095 10.1038/srep20250 PMC4735296

[CR6] Xu H, Russell SN, Steiner K, et al. Targeting PI3K-gamma in myeloid driven tumour immune suppression: a systematic review and meta-analysis of the preclinical literature[J]. Cancer Immunol Immunother. 2024;73(10):204. 39105848 10.1007/s00262-024-03779-2 PMC11303654

[CR28] Yang CS, Melhuish TA, Spencer A, et al. The protein kinase C super-family member PKN is regulated by mTOR and influences differentiation during prostate cancer progression[J]. Prostate. 2017;77(15):1452–67. 28875501 10.1002/pros.23400 PMC5669364

[CR7] Zeng W, Liu H, Mao Y et al. Myeloid–derived suppressor cells: key immunosuppressive regulators and therapeutic targets in colorectal cancer (review)[J]. Int J Oncol, 2024, 65(3). 10.3892/ijo.2024.5673 PMC11299769 39054950

[CR26] Zhang Y, Li Z, Huang Y, et al. Advancements in immunotherapy for advanced esophageal squamous cell carcinoma: a comprehensive review of current strategies and future directions[J]. Expert Rev Clin Immunol. 2024;20(8):971–84. 38884604 10.1080/1744666X.2024.2368194

