# Supplementary material for: PKN2 enhances the immunosuppressive activity of polymorphonuclear myeloid-derived suppressor cells in esophageal carcinoma by mediating fatty acid oxidation
Source: Mol Med. 2025 Mar 11;31:92. doi: 10.1186/s10020-025-01132-6 (PMC11900251; doi:10.1186/s10020-025-01132-6)
Supplement: Supplementary file 1 — Supplementary Material 1 [file 10020_2025_1132_MOESM1_ESM.docx]

**Supplemental Method Details**

***Isolation of macrophages, B cells, monocytes, plasma cells, and MDSCs from mice***

C57BL/6 male mice aged 6-8 weeks were purchased from Changzhou Cavens Laboratory Animal Co. (Changzhou, Jiangsu, China). The mice were administered 1 mL of 3% mercaptoacetate broth via intraperitoneal injection once daily for a period of three consecutive days. Following a three-day period, the mice were euthanized. The mice were immersed in 75% ethanol for a period of 3-5 minutes, after which they were transferred to an ultraclean table and fixed in the supine position. A small incision was made along the abdominal wall, and the entire abdominal skin was subsequently excised. Subsequently, the skin on both sides was pinched and gradually torn to expose the peritoneum. In the right lower abdomen, 5 mL of pre-cooled phosphate buffer saline (PBS) was slowly injected into the abdominal cavity, with gentle rubbing of the mice's abdomens, and left to stand for 3-5 minutes to allow for optimal distribution of the liquid within the abdominal cavity. Subsequently, the fluid within the abdominal cavity was extracted and transferred to a centrifuge tube. The collected fluid was subjected to centrifugation at 1000 rpm for 5 minutes at room temperature, after which the supernatant was discarded. The cell precipitate was resuspended and inoculated with RPMI-1640 culture medium, and the culture system was incubated at 37°C for a period of 2-3 hours. The non-adherent cells were removed by washing, and the remaining adherent cells were identified as macrophages.

Mouse blood was collected from healthy mice and lymphocytes were isolated from blood samples using the Mouse Peripheral Blood Lymphocyte Separation Kit (No. C0029S, Beyotime, Beijing, China), following the manufacturer’s instructions. Subsequently, mouse B cells were isolated from lymphocytes using magnetic-activated cell separation (MACS). Briefly, mouse CD19^+^ microbeads (Miltenyi Biotec Inc., Auburn, CA, USA) were added to the samples, which had been previously resuspended. During the incubation period, the MidiMACS Sorting Magnet was mounted on the MACS Multi Rack, and the LS sorting column was placed into the magnet with a sterile 15 mL centrifuge tube positioned beneath the sorting column. The incubation period should be terminated by the addition of 10 mL of pre-cooled sorting buffer at 4°C. Subsequently, the beads were suspended and subjected to centrifugation at 4°C (200 g for 10 minutes). During the centrifugation process, the LS column was rinsed with 3 mL of pre-cooled sorting buffer at 4°C. Upon completion of the rinse, a new sterile 15-mL centrifuge tube was positioned beneath the column. Subsequently, the washings were discarded, and the cells were resuspended at a concentration of 100 × 10⁶/500 μL. The cells were then introduced to the LS column and 3 mL of sorting buffer was added for the purpose of rinsing the column three times. Subsequently, the LS sorting column was removed from the magnet and placed in a new sterile 15-mL centrifuge tube. An additional 5 mL of sorting buffer was added to the column, after which the plunger was inserted and compressed to elute the positive cells, which were B cells. Subsequently, the B cells were collected via centrifugation at 200 g for 10 minutes. The supernatant was removed, and the precipitate was resuspended in RPMI 1640 culture medium containing 10% fetal bovine serum (FBS) for subsequent cell culture.

Mouse monocytes were isolated from blood using the EasySep™ Mouse Monocyte Isolation Kit (No. 19861, STEMCELL Technologies Inc., Vancouver, BC, Canada), following the manufacturer’s instructions.

Mouse plasma cells were isolated from bone marrows using the CD138^+^ Plasma Cell Isolation Kit (No. 130-092-530, Miltenyi Biotechnology, Bergisch Gladbach, Germany), following the manufacturer’s instructions.

MDSCs were isolated from mouse bone marrows. In order to expose the femur and tibia, it is necessary to excise the hind leg of the mouse above the hip joint. Subsequently, the surrounding muscle and tissue was excised. Subsequently, the cleaned bones were immersed in a petri dish containing 70% ethanol for a period of 5-10 seconds, thereby ensuring complete external sterilization. All bones were cleaned and placed in a sterile tube surrounded by ice. The extremities of the bones were trimmed with scissors. A syringe was filled with precooled RPMI Complete Medium (R10), and the syringe needle was inserted into the bone to facilitate the rinsing of the bone marrow into the surrounding centrifuge tube with ice. This process was repeated two to three times until the bone is completely white. The bone marrow fluid was aspirated on multiple occasions with a pipette to disrupt any clumps that may have formed. Centrifuge at 1,500 rpm for 5 minutes, repeating the process one to two times. The cells were diluted with 10 mL of R10 + 20 ng/mL GM-CSF and inoculated into culture dishes at a density of 2 × 10⁶ live cells/dish. The dishes were incubated in a 37°C incubator with 5% CO₂ for the duration of the experiment. On the third day, an additional 10 mL of R10 + 20 ng/mL GM-CSF was incorporated into the existing solution. On the sixth day, half of the medium was removed. The removed medium was briefly centrifuged, and the cell precipitate was resuspended in 10 mL of fresh R10 + 20 ng/mL GM-CSF, after which it was returned to the original culture dish. The non-adherent and loosely adherent cells present in the culture supernatant can be harvested by gently rinsing with PBS and subsequently pooled for use in subsequent experiments.

***Quantitative real-time PCR (qRT-PCR)***

Total RNA was isolated using TRIzol reagent (Invitrogen, Waltham, MA, USA), in accordance with the protocol specified by the manufacturer for tissue samples. Subsequently, cDNA was synthesized from total RNA using the QuantiTect Reverse Transcription kit (Qiagen, Manchester, UK) in accordance with the manufacturer's instructions. To assess the mRNA expressions, qRT-PCR was performed using the IQ SYBR Green Supermix (Bio-Rad, Hercules, CA, USA) and analyzed on the IQ5 Thermocycler (Bio-Rad, Hercules, CA, USA). The mRNA expressions were normalized to that of the housekeeping gene, GAPDH, with qRT-PCR assays performed in triplicate. The sequences of the primers utilized in qRT-PCR are provided below for reference. PKN2 forward primer 5’-CGACCAAAACTCCAAAGACA-3’ and reverse primer 5’- GTCTTCCCCAAGTGGCAATA-3’; TECR forward primer 5’-GGTGGAGATTCGGGATGCAA-3’ and reverse primer 5’- GGGACTTCCCCTGTGTCTTG-3’; CPT1B forward primer 5’- TCTAGGCAATGCCGTTCAC-3’ and reverse primer 5’- GAGCACATGGGCACCATAC-3’; ACAT1 forward primer 5’- AGCTGTTTCTCTGGGCCATC-3’ and reverse primer 5’- CCTCCTCCTCCGTTGCAAAT-3’; HADH forward primer 5’- TGCATTTGCCGCAGCTTTAC-3’ and reverse primer 5’- GTTGGCCCAGATTTCGTTCA-3’; CPT2 forward primer 5’- CCCAAACCCAGTCGTGATGA-3’ and reverse primer 5’- GCCCAGACATCTCGGTTCTC-3’; MCAT forward primer 5’- GGTACTGGGCTACGATCTGC-3’ and reverse primer 5’- AGTTGTCGATGACCGCCG-3’; ACADM forward primer 5’- ATGCCTGTGATTCTTGCT3’ and reverse primer 5’- TAACATACTCGTCACCCTTC-3’; ECHS1 forward primer 5’- CACTGTTGTCCTCAGTCCGC-3’ and reverse primer 5’- GGTCTCCAGTGCTTGGTTGA-3’; OXSM forward primer 5’- GATCCTAAGCTAGCCTGCCG-3’ and reverse primer 5’- AAAGCACCTTCTCCTTCGGG-3’; GAPDH forward primer 5’- TCACCACCATGGAGAAGGC-3’ and reverse primer 5’- GCTAAGCAGTTGGTGGTGCA-3’. Gene expression levels were determined using the 2^−ΔΔCt^ method to calculate fold changes, where ΔΔCt is calculated as ΔCt of the sample minus ΔCt of the control, with ΔCt being the cycle threshold (Ct) value of the target gene minus that of the housekeeping gene.

**Western blotting**

A total of approximately 30 µg of protein samples were subjected to sodium dodecyl sulfate-polyacrylamide gel electrophoresis (SDS-PAGE) on a 10% gel. Subsequently, the proteins were transferred onto polyvinylidene difluoride (PVDF) membranes (Millipore, Temecula, CA, USA). Subsequently, the membranes were incubated for one hour at room temperature with 10% non-fat dry milk in TRIS-buffered saline with 0.5% Tween 20 (TBST). Subsequently, the samples were incubated with specific primary antibodies at 4 ℃ overnight. The antibodies utilized included anti-PKN2 (A19746, ABclonal, Wuhan, Hubei, China; MA5-15887, Invitrogen, Waltham, MA, USA), anti-Arg-1 (A4923, Abclonal Technology, Wuhan, Hubei, China), TECR (PA5-100691, Invitrogen), CPT1B (ab134988, Abcam), ACAT1 (ab307597, Abcam), HADH (ab154088, Abcam), CPT2 (ab110293, Abcam), MCAT (ab235036), ACADM (ab92461, Abcam), ECHS1 (ab228631, Abcam), OXSM (ab229111, Abcam), p-STAT3 (ab32143, Abcam), STAT3 (ab68153, Abcam), COX IV (ab202554, Abcam), and anti-β-tubulin (ab7291, Abcam). Subsequently, the membranes were incubated with secondary antibodies for an additional hour at room temperature, diluted at a ratio of 1:5000 in TBST. The immunoblots were detected using an enhanced chemiluminescence (ECL) kit sourced from Pierce in Shanghai, China. The bound antibodies were visualized using the FluorChem FC3 system from ProteinSimple in the USA, and the band densities were quantified with the AlphaView software, version 3.4.0.0. The intensities of the proteins were normalized against β-tubulin for ratio calculation.

**Supplemental Figures**


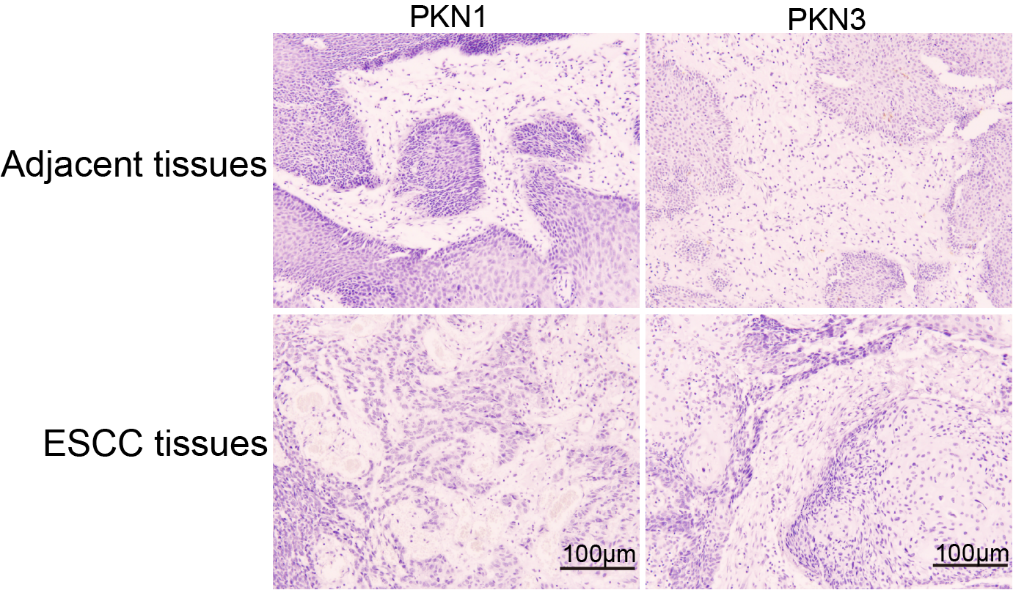


Fig. S1. Immunohistochemistry of PKN1 and PKN3 antibodies for EC tissues and adjacent tissues.


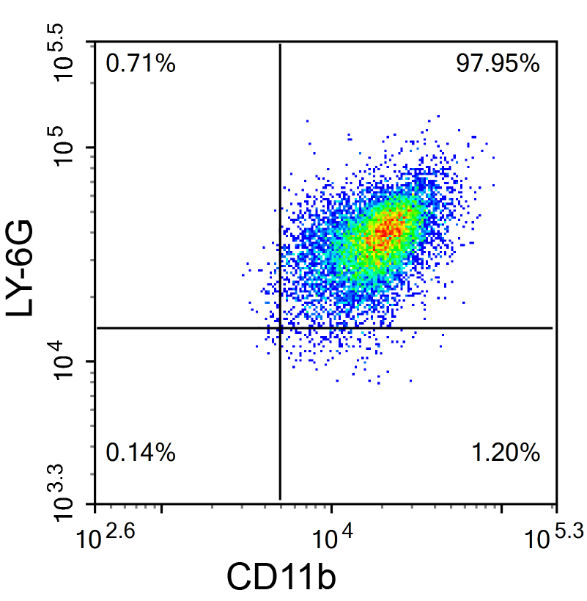


Fig. S2. PMN-MDSCs were isolated from EC mouse spleen tissues and identified by flow cytometry with 97.95% of CD11b^+^LY-6G^+^.


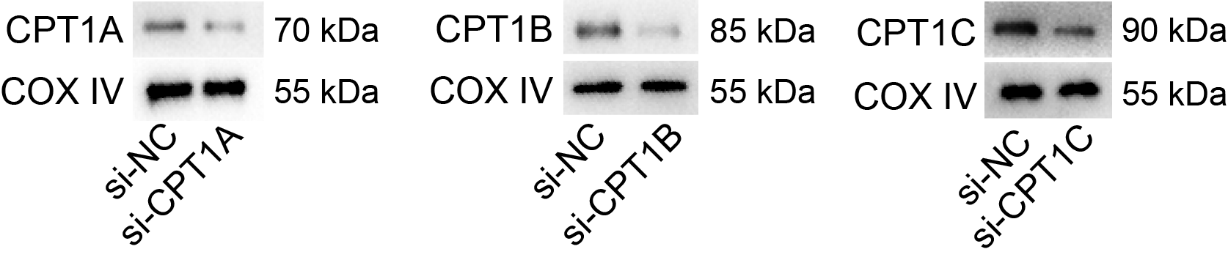


Fig. S3. We silenced CPT1A, CPT1B, and CPT1C in PKN2-highly expressed PMN-MDSCs, respectively, by using siRNAs and confirmed the transfection efficiency by Western blotting.


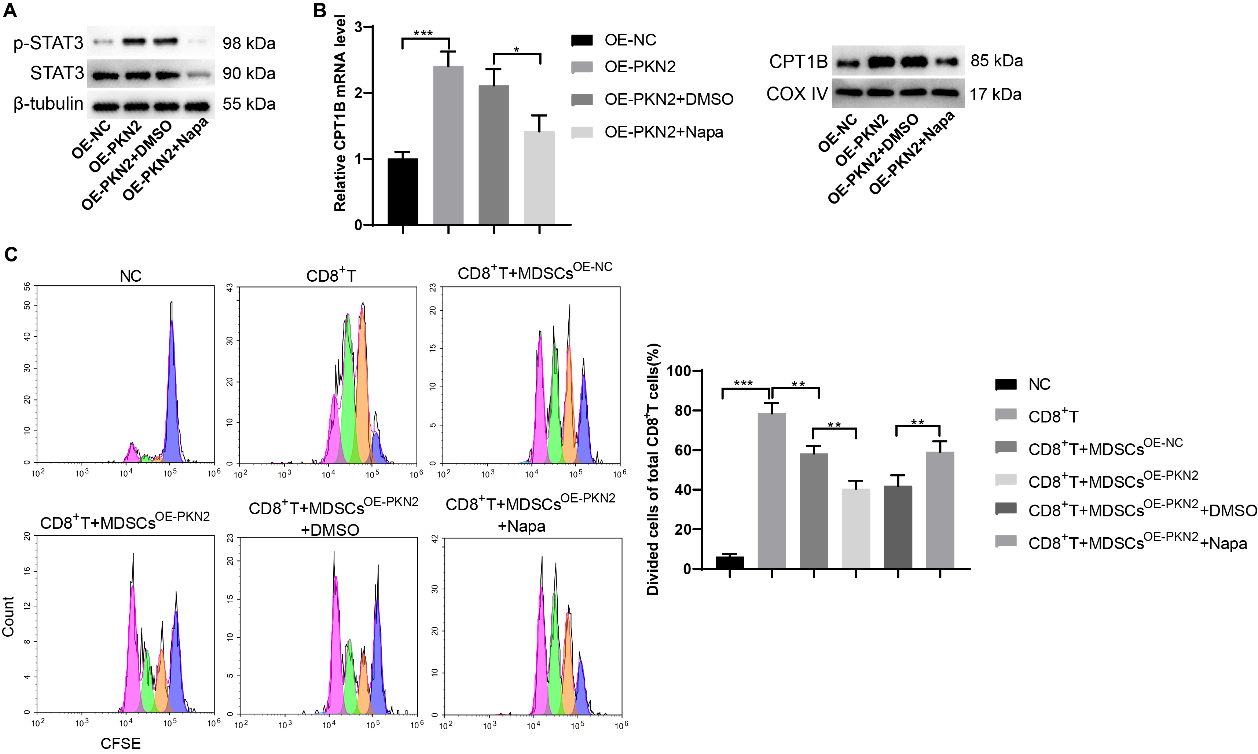


Fig. S4. STAT3 inhibitor Napabucasin inhibits the promotion of CPT1B expression and MDSC immunosuppressive capacity by PKN2. PKN2 plasmid was transfected with MDSC with Napabucasin (Napa, 1 µM), 0.01% DMSO as control. (B) STAT3 and CPT1B expression was detected 24 h after transfection, and the results showed that Napabucasin treatment decreased STAT3 protein and its phosphorylation level. In addition, Napabucasin abolished the promoting effect of PKN2 on CPT1B expression. Statistical analysis: one-way ANOVA with Tukey’s post-hoc test. **P* < 0.05. ****P* < 0.001. (C) MDSCs were co-cultured with T cells by the same method as Figure 3. The results showed that Napabucasin abolished the effect of PKN2 on MDSC immunosuppressive capacity. Statistical analysis: one-way ANOVA with Tukey’s post-hoc test. ***P* < 0.01. ****P* < 0.001.
